# Hnf-1β Transcription Factor Is an Early Hif-1α-Independent Marker of Epithelial Hypoxia and Controls Renal Repair

**DOI:** 10.1371/journal.pone.0063585

**Published:** 2013-05-21

**Authors:** Stanislas Faguer, Nicolas Mayeur, Audrey Casemayou, Anne-Laure Pageaud, Claire Courtellemont, Claire Cartery, Gilbert J. Fournie, Joost P. Schanstra, Ivan Tack, Jean-Loup Bascands, Dominique Chauveau

**Affiliations:** 1 Département de Néphrologie et Transplantation d’organes, CHU Rangueil, Toulouse, France; 2 Institut National de la Santé et de la Recherche Médicale (INSERM), U1048, Institut des maladies métaboliques et cardiovasculaires (équipe 12), Toulouse, France; 3 Centre de référence des maladies rénales rares, CHU Rangueil, Toulouse, France; 4 Service des explorations physiologiques, CHU Rangueil, Toulouse, France; 5 Département d’Anesthésie - Réanimation, équipe Accueil Inserm 4564, CHU Purpan, Toulouse, France; 6 Université Toulouse III Paul-Sabatier, Toulouse, France; University of Cincinnati, United States of America

## Abstract

Epithelial repair following acute kidney injury (AKI) requires epithelial-mesenchyme-epithelial cycling associated with transient re-expression of genes normally expressed during kidney development as well as activation of growth factors and cytokine-induced signaling. In normal kidney, the Hnf-1β transcription factor drives nephrogenesis, tubulogenesis and epithelial homeostasis through the regulation of epithelial planar cell polarity and expression of developmental or tubular segment-specific genes. In a mouse model of ischemic AKI induced by a 2-hours hemorrhagic shock, we show that expression of this factor is tightly regulated in the early phase of renal repair with a biphasic expression profile (early down-regulation followed by transient over-expression). These changes are associated to tubular epithelial differentiation as assessed by KSP-cadherin and megalin-cubilin endocytic complex expression analysis. In addition, early decrease in *Hnf1b* expression is associated with the transient over-expression of one of its main target genes, the suppressor of cytokine signaling *Socs3*, which has been shown essential for renal repair. *In vitro*, hypoxia induced early up-regulation of Hnf-1β from 1 to 24 hours, independently of the hypoxia-inducible factor Hif-1α. When prolonged, hypoxia induced Hnf-1β down-regulation while normoxia led to Hnf-1β normalization. Last, Hnf-1β down-regulation using RNA interference in HK-2 cells led to phenotype switch from an epithelial to a mesenchyme state. Taken together, we showed that Hnf-1β may drive recovery from ischemic AKI by regulating both the expression of genes important for homeostasis control during organ repair and the state of epithelial cell differentiation.

## Introduction

In the general population, acute renal failure (ARF) is noted in 1% of all hospital admissions. Hospital-acquired ARF occurs in up to 7% of all hospitalizations. Ischemic acute kidney injury (AKI) is the major cause of ARF and is associated with increased mortality in hospitalized populations [1]. Renal pathological changes of ischemic AKI are characterized by changes in proximal tubule epithelium. Disruption of the cytoskeleton and the loss of cell polarity result in the loss of the proximal tubule brush border, patchy loss of tubule cells, focal areas of proximal tubular dilatation and distal tubular casts, and areas of cellular regeneration characterized by cell proliferation [2]. Following AKI, replacement of lethally injured tubular cells is achieved by a complex reparative process leading to cell proliferation. Epithelial-mesenchyme-epithelial cycling allows almost complete recovery of renal architecture and renal function in most of the cases [3]. Tight regulation of this complex process involves cell cycle control [4], transient re-expression of genes normally expressed during kidney development, including *Pax2*, *Notch-2*, *Wnt4* and *Ets-1* [5], [6], [7], [8], and transient activation of growth factors- or cytokine-induced signaling, including the hepatocyte and epidermal growth factor (HGF/EGF) and the interleukine-6 receptor pathways [9], [10].

During early kidney development, hepatocyte nuclear factor-1β (Hnf-1β) drives nephrogenesis, tubulogenesis and epithelial maturation through the regulation of epithelial planar cell polarity and expression of developmental or tubular segment-specific genes [11], [12], [13]. *Hnf1b* is expressed during early mouse embryogenesis, especially in the Wolffian duct, the ureteric bud and the metanephric kidney [14]. During later stages of kidney development, *Hnf1b* is expressed in all segments of the nephron, from the proximal tubule (PT) to the collecting duct. Hnf-1β is a transcription factor that controls the expression of a number of genes including *Pkhd1*, *Umod* and *Socs3* [15], [16]. Hnf-1β acts as a homo- or heterodimer with the closely related transcription factor Hnf-1α. The latter is mostly expressed in PT cells where it drives cells differentiation [17]. Antenatal kidney-specific conditional inactivation of *Hnf1b* in mice induces polycystic kidney disease with lethal renal failure around three weeks after birth [15], [18]. Some recent studies suggested that Hnf-1β may have a role in epithelial kidney and liver repair [19], [20].

Data concerning the role of Hnf-1β in renal repair following AKI are scarce. Interestingly, invalidation of *Hnf1b* after the end of renal development (*i.e.* after P10 in mouse) is not followed by renal changes, except when cells are forced to enter the cell cycle [19]. In mice with renal specific *Hnf1b* invalidation after P10, ischemic AKI promotes tubular dilatation and cystic kidney disease. Among Hnf-1β target genes *Socs3* is a key player in epithelial repair following ischemic AKI. Within the first hours following ischemic injury, a dramatic increase in the intra-renal expression of *Socs3*, a suppressor of cytokine signaling, induced through an interleukine-6-mediated feedback, has been identified [21]. Reduced *Socs3* expression in proximal tubular cells accelerates acute renal failure [22]. In addition, it has been demonstrated that *Socs3* negatively regulates signaling of various growth factors and cytokines, including EGF, leukemia inhibitor factor, fibroblast growth factor, angiotensin-II and insulin-like growth factor-1, all involved in renal repair [23], [24], [25], [26].

Surprisingly, expression of *Hnf1b* during early steps of renal repair has not been studied. We thus investigated the expression of *Hnf1b* in parallel with some target genes in an ischemic AKI model. We found that Hnf-1β drives recovery from ischemic AKI by regulating both the expression of important genes for homeostasis control during PT repair, and the state of epithelial cell differentiation. In addition, we deciphered the respective roles of the hypoxia-inducible factor Hif-1α up-regulation and low oxygen pressure per se in the regulation of the *Hnf1b* expression.

## Results

### Assessment of AKI in a Mouse Model of Hemorrhagic Shock

**Figure 1:**
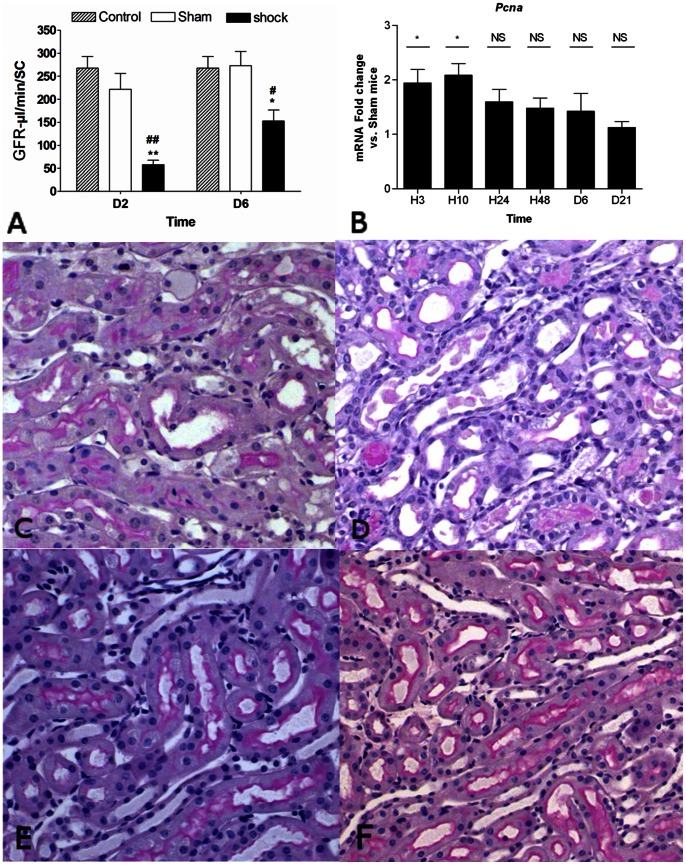
Renal functional, histological and mRNA expression changes after a 2 hours-hemorrhagic shock in mouse. *A*. GFR assessed by inulin clearance at 2 days and 6 days after a 2-hours hemorrhagic shock. Significant decrease of GFR was detected at day 2 and day 6 in shock compared to sham mice; *B*, Sequential whole-kidney expression of *Pcna* normalized to *Gapdh* mRNA amount at 3 hrs, 10 hrs, 24 hrs, 48 hrs, 6 days and 21 days (H3, H10, H24, H48, D6 and D21, respectively) after hemorrhagic shock. Data are shown as ratio of mRNA expression between shock and sham mice. *P<0.05, **P<0.01 shock (n = 5) vs. sham (n = 4); *C–F*, Renal injury after hemorrhagic shock (sham mice (*E–F*); shock mice (*C–D*)) at day 2 and day 6 (PAS coloration, ×400). *GFR*, glomerular filtration rate; *NS*, not significant.

We used a recently developed mouse model of AKI induced by a 120-minutes hemorrhagic shock-related hypotension, as previously described [27]. In this model, renal damages were confirmed by determining functional, histological and mRNA expression changes of key AKI genes. At day 2 and 6, a significant decrease of the glomerular filtration rate was observed in shocked mice (Fig. 1a). Periodic acid-Schiff and Masson’s trichrome staining of kidney sections from shocked mice showed typical features of AKI, including disruption of the epithelial brush border, flattening of the epithelia and tubular casts, while these histological changes were not observed in sham mice (Fig. 1c–f). Consistent with previous mouse models using an ischemia/reperfusion (I/R) model to mimic AKI [21], [28], assessment of cell proliferation by *Pcna* mRNA expression showed a significant increase within the first 10 hours (Fig. 1b).

Thus a 120-minutes hemorrhagic shock resulted in significant AKI with dramatic functional, histological and mRNA expression changes of key AKI genes, and may be a valuable tool to decipher the mechanisms of renal repair.

### Renal Expression of *Hnf-1β* and Some of its Target Genes after Ischemic AKI in Mouse

**Figure 2:**
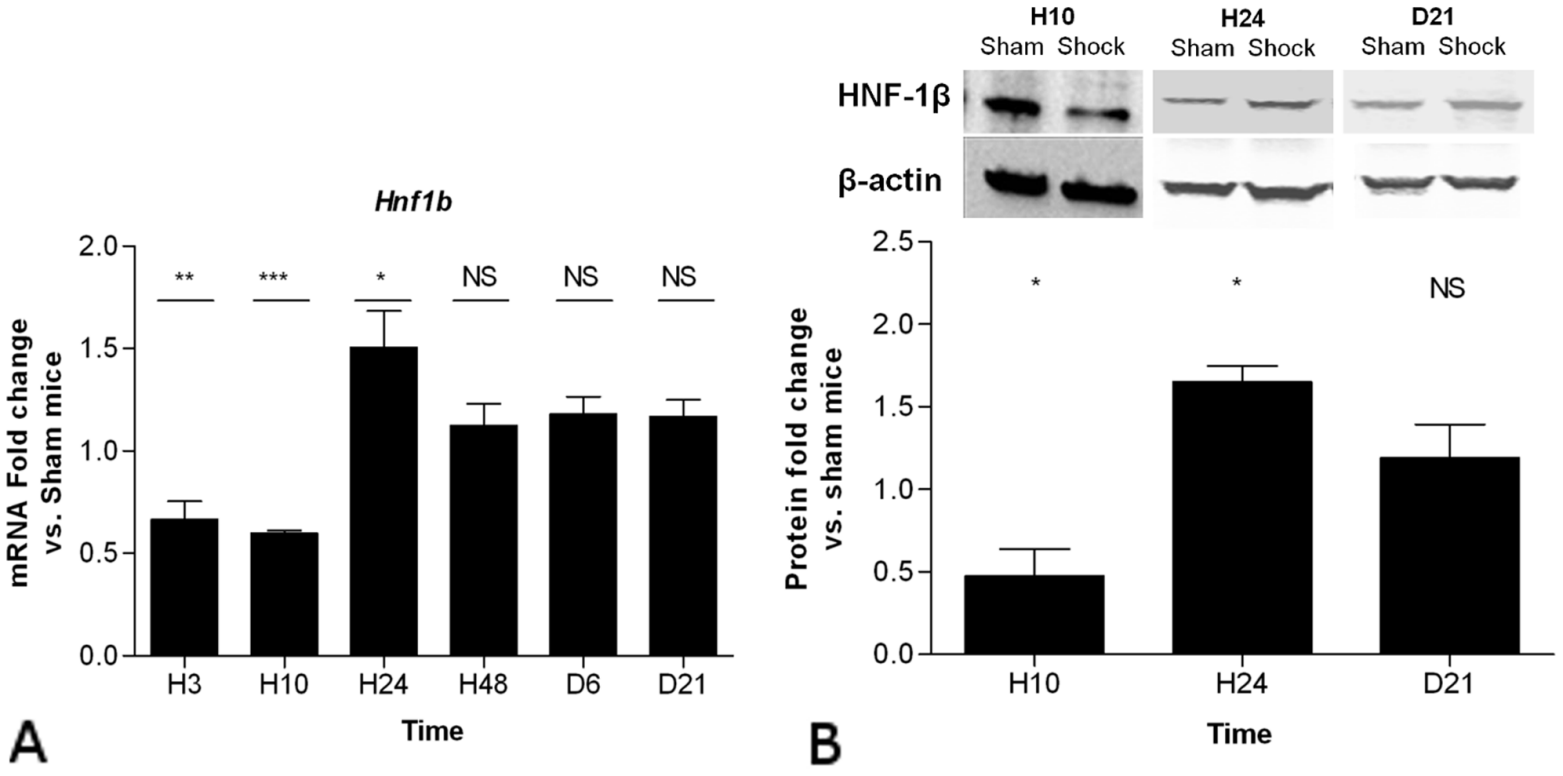
Sequential whole-kidney expression of *Hnf1b* after hemorrhagic shock. *A,* Sequential whole-kidney expression of *Hnf1b* normalized to *Gapdh* mRNA amount at 3 hrs, 10 hrs, 24 hrs, 48 hrs, 6 days and 21 days (H3, H10, H24, H48, D6 and D21, respectively) after hemorrhagic shock. B, whole-kidney expression of Hnf-1β (protein) normalized to Beta-Actin amount at 10 hrs, 24 hrs and 21 days after hemorrhagic shock. Data are shown as ratio of mRNA or protein expression between shock and sham mice. *P<0.05, **P<0.01; shock (n = 5) vs. sham (n = 4); *NS*, not significant.

In this mouse model of hemorrhagic shock-induced AKI, we now show a significant ∼50% decrease in the expression of *Hnf1b* within the first 10 hours post-shock followed by a transient over-expression at 24 hours (Fig. 2a). The kinetics of Hnf-1β expression was confirmed at protein level (Fig. 2b).

**Figure 3:**
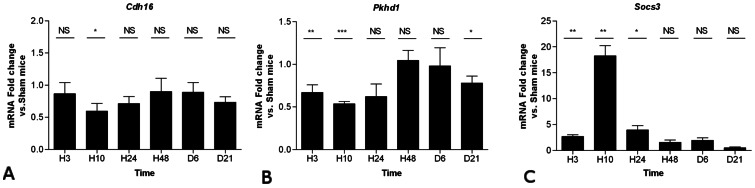
Sequential whole-kidney expression of *Cdh16*, *Pkhd1* and *Socs3* after hemorrhagic shock. Relative mRNA expression of *Cdh16* (A), *Pkhd1* (B) and *Socs3* (C) at 3 hrs, 10 hrs, 24 hrs, 48 hrs, 6 days and 21 days (H3, H10, H24, H48, D6 and D21, respectively) normalized to *Gapdh* mRNA. Data are shown as ratio of mRNA (or protein) expression between shock and sham mice *P<0.05, **P<0.01; shock (n = 5) vs. sham (n = 4); *NS*, not significant.

Renal expression of *Cdh16* (KSP-cadherin), *Pkhd1* (Polyductin) *and Socs3*, three genes directly regulated by Hnf-1β, were also assessed. *Cdh16* and *Pkhd1* are known to be positively regulated, while *Socs3* is negatively regulated by Hnf-1β [15], [16]. A significant decrease of *Cdh16* and *Pkhd1* expression was observed 10 hours after the hemorrhagic shock followed by progressive normalization until day 21 (Fig. 3a–b). Conversely, the expression of *Socs3* (a gene negatively regulated by Hnf-1β) displayed a mirror expression profile with Hnf-1β in this model (Fig. 3c). These results suggest that the expression of Hnf-1β and three of its target genes is tightly regulated during the regeneration phase following ischemic AKI in mouse.

### Renal Expression of Hnf1a and Proximal Tubule Markers after Ischemic AKI in Mouse

**Figure 4:**
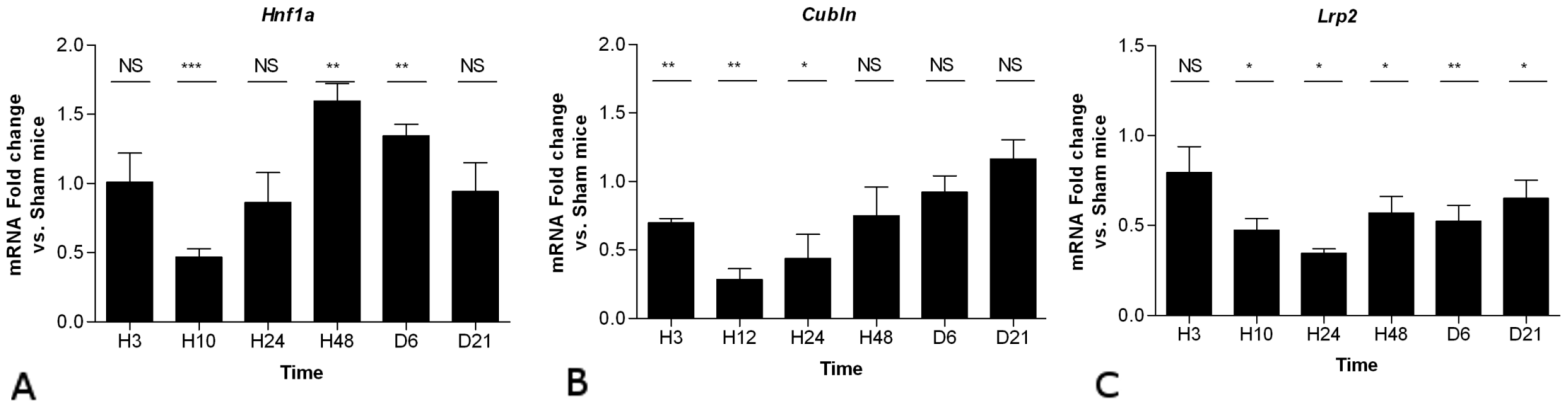
Sequential whole-kidney expression of *Hnf1a* and the *Lrp2* and *Cubln* megalin-cubilin endocytic complex after hemorrhagic shock. Relative mRNA expression of *Hnf1a*, *Lrp2* and *Cubln* at 3 hrs, 10 hrs, 24 hrs, 48 hrs, 6 days and 21 days (H3, H10, H24, H48, D6 and D21, respectively) normalized to *Gapdh* mRNA. Data are shown as ratio of mRNA expression between shock and sham mice. *P<0.05, **P<0.01; shock (n = 5) vs. sham (n = 4); *NS*, not significant.

Hnf-1α is involved in epithelial repair in the liver [28]. In addition, Hnf-1α is required for proper differentiation of renal proximal tubule [17] and acts in homo- or heterodimers with Hnf-1β, but respective target genes of Hnf-1β - Hnf-1β and Hnf-1β - Hnf-1α dimers have not been fully characterized. We have therefore studied *Hnf1a* renal expression after ischemic AKI in parallel with *Hnf1b*. A significant ∼50% decrease in the expression of *Hnf1a* was observed at 10 hours followed by a transient rebound at 48 hours (Fig. 4a). Thus, temporally changes in *Hnf1a* expression occur later than the change in *Hnf1b* expression. We also studied the renal expression of proximal tubule markers *Lrp2* and *Cubln*, which encode for the endocytosis complex megalin-cubilin, in this ischemic AKI mouse model. Expression of both genes was significantly decreased from 3 up to 24 hours after hemorrhagic shock. Expression of *Cubln* normalized at 48 hours while *Lrp2* remained down regulated until 21 days after the insult (Fig. 4b–c). Persistent down-regulation of *Lrp2* at day 21 suggested permanent proximal tubule dysfunction escaping clinical and pathological detection.

### *In vitro* studies of *Hnf1b* and Hif-1α Expression Under Hypoxic Conditions

**Figure 5:**
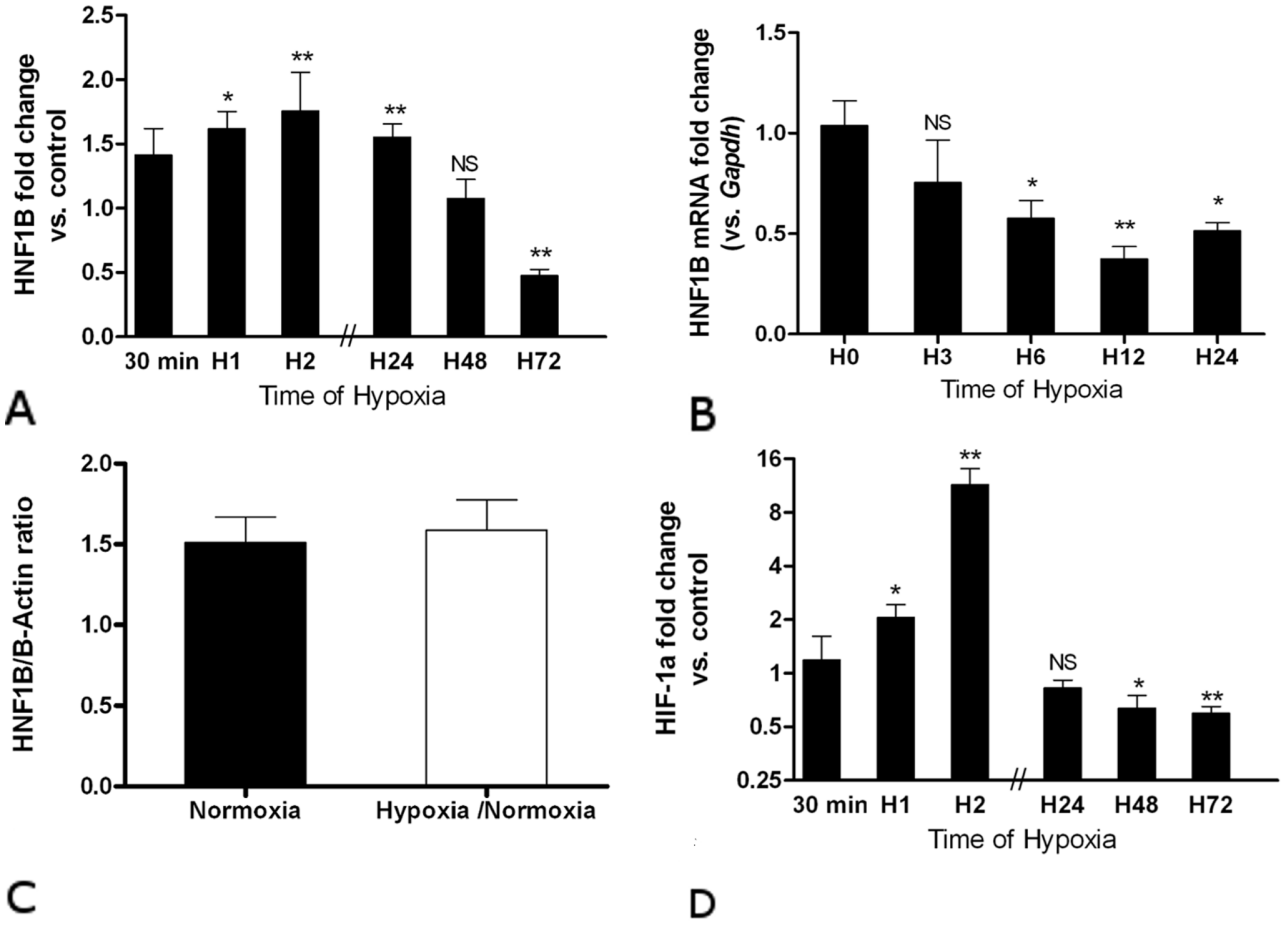
Expression of Hnf-1β and Hif-1α in HK-2 cells under hypoxic conditions. Relative protein expression of Hnf-1β protein (A) and mRNA (B), and Hif-1α (D) after 30 min, 1 hr, 2 hrs, 24 hrs, 48 hrs or 72 hrs of hypoxia. (C) Relative expression of Hnf-1β after 2 hours of hypoxia followed by 24 hours of normoxia. Relative expression of protein expression was normalized to Beta-actin. Data are shown as ratio of protein expression between cells grown under normoxic (n = 6) or hypoxic condition (n = 6). *P<0.05, **P<0.01; *NS*, not significant.

To decipher the molecular mechanisms that controls *Hnf1b* expression in kidney during and after ischemic injury, we assessed the consequences of hypoxia on Hnf-1β *in vitro.* HK-2 cells (a cell line issued from human proximal tubule) were grown under normoxic (21% oxygen) or hypoxic (1% oxygen) conditions, and Hnf-1β levels were measured at various time of hypoxia. Western blot analyses showed a significant increase of Hnf-1β expression as early as one hour after hypoxia and up to 24 hours (Fig. 5a and Fig. S1), followed by a progressive decline reaching control values after 48 hours hypoxia and a down regulation at 72 hours. Restoring normoxia after 2 hours of hypoxia leads to the normalization of Hnf-1β expression at 24 hours (Fig. 5c). Contrasting with changes of Hnf-1β protein, *Hnf1b* mRNA was significantly decreased during hypoxia (Fig. 5b). Hence, these data suggest that Hnf-1β amount in epithelial cells submitted to hypoxia is controlled at both protein (early effect) and mRNA (late effect) level.

**Figure 6:**
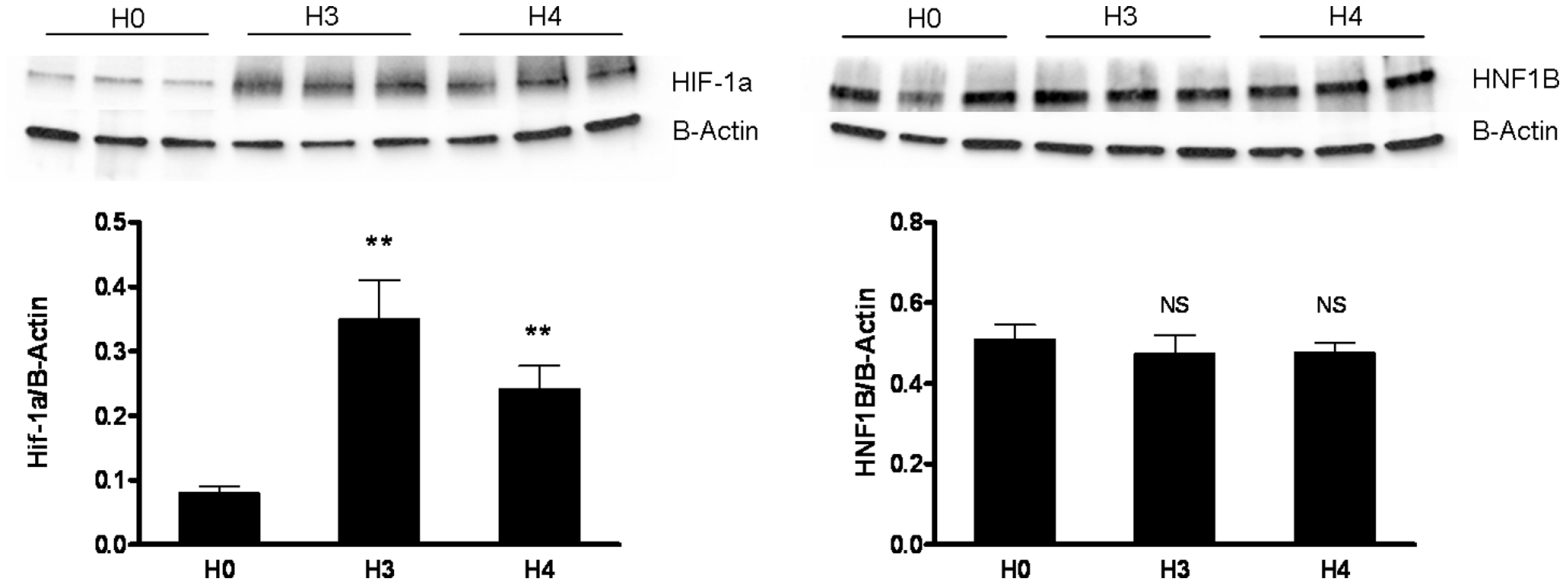
Expression of Hnf-1β and Hif-1α in HK-2 cells incubated with Hif-1α-specific prolylhydroxylase inhibitor. Relative protein expression of Hif-1α (A) and Hnf-1β (B) after 3 and 4 hours of exposure to Hif-1α specific prolyl hydroxylase inhibitor. Protein expressions were normalized to Beta-actin (n = 6). **P<0.01; *NS*, not significant.

The transcriptome adaptive response to hypoxia has been shown to be primarily controlled by hypoxia-inducible factor Hif-1α [29]. To determine whether *Hnf1b* expression is dependant of Hif-1α we analyzed its expression in HK-2 cells submitted to hypoxia (see raw data of western blot in supplementary material online). As shown in figure 5d, Hif-1α was early and significantly up-regulated when HK-2 cells were submitted to 1%-hypoxia reaching a maximal value after 2 hours of hypoxia. This increase was transient since persistent hypoxia lead to down-regulation of Hif-1α after 24 hours of hypoxia. This down regulation became highly significant at 72 hours of hypoxia. As Hnf-1β and Hif-1α expression followed a similar but not overlapping expression profile with time in hypoxic HK-2 cells, we hypothesized that the increase in Hnf-1β could be related to Hif-1α overexpression. To test it, we cultivated HK-2 cells in normoxic conditions and added to the culture medium an inhibitor of Hif-1α specific prolylhydroxylases (PHD) to inhibit Hif-1α degradation. As expected, PHD inhibition was accompanied by a significant over-expression of Hif-1α (Fig. 6a and Fig. S2), while Hnf-1β expression was not modified (Fig. 6b), a finding consistent with a differentially targeted regulation for each of the transcription factor under hypoxia. In line with this result, we failed to find HIF-1α binding region ([A/G]CGTG motif; *i.e.* hypoxia responsive element), conserved in at least four species [30] (Homo Sapiens, Mus Musculus, Bos Taurus and Equus Caballus), in the 4000 pb upstream of the transcription start site of *Hnf1b*.

### Role of Hnf-1β in Epithelial Cells Submitted to Hypoxia

**Figure 7:**
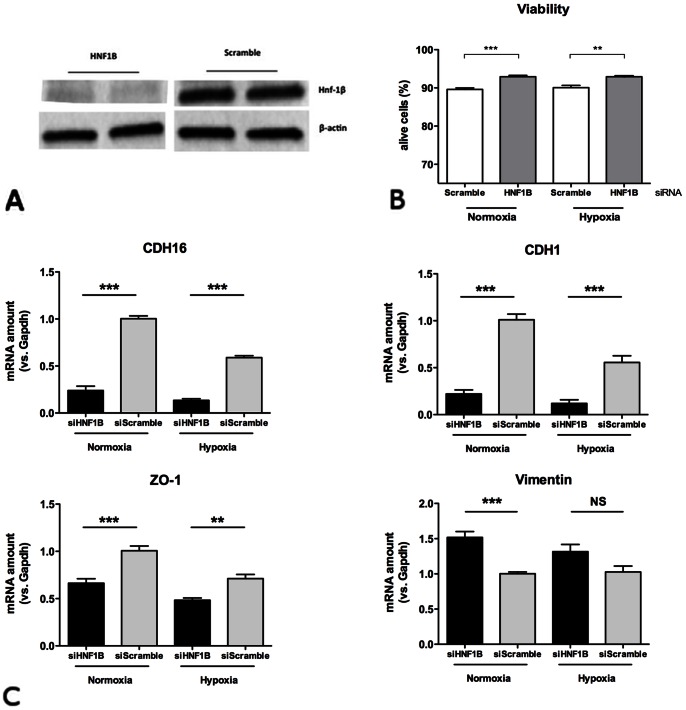
Molecular consequences of Hnf-1β inhibition in epithelial cells submitted to hypoxia. (A) Amount of Hnf-1β protein in HK-2 cells after 48 hours of specific siRNA interference targeting HNF1B gene. (B) Percentage of HK-2 cells alive (by FACS analysis) after 24 hours of normoxia or hypoxia following transfection with control siRNA (siScramble) or *HNF1B*-specific siRNA (siHNF1B). (C) mRNA expression of *CDH1*, *CDH16*, *ZO-1* and *VIMENTINE* (normalized to GAPDH mRNA) after 24 hours of hypoxia, depending of *HNF1B* expression. Groups were compared using one-way ANOVA test followed by Bonferroni’s correction (n = 4–7). **P<0.01; ***P<0.001.

To assess the role of Hnf-1β during hypoxia, HK-2 cells were efficiently transfected with specific small interference RNA (siRNA)-targeting *HNF1B* (Figure 7A) and then grown under normoxic or hypoxic conditions for 24 hours. Using Fluorescence-activated cell sorting (FACS) approach, we observed that viability was slightly but significantly higher in HK-2 cells with previous *HNF1B* down-regulation (Figure 7B). Moreover, transfection with *HNF1B*-specific siRNA was followed by dramatic phenotypic changes of HK-2 cells, in both normoxic and hypoxic conditions, with significant down-regulation of epithelial markers E-Cadherin, KSP-Cadherin and ZO-1, and a trend in up-regulation of the mesenchyme marker Vimentin (Figure 7C).

## Discussion

Hnf-1β is a transcription factor that controls planar cell polarity, tubulogenesis and tubular segment differentiation [11], [12], [13], [15], which are all events required for proper renal repair. To date, the role of Hnf-1β in epithelial regeneration remains highly elusive. The main finding in this study is that *Hnf1b* mRNA and protein expression is tightly regulated during epithelial repair following ischemic AKI induced by a 120-minutes hemorrhagic shock-related hypotension. The biphasic kinetics suggests that *Hnf1b* may control expression of molecular actors required for proper epithelial repair (e.g. *Socs3*). However, injury of tubules in ischemic AKI happens in both ischemic and reperfusion periods. In our model of hemorrhagic shock, tubular injuries were identified up to 6 days after the insult. Hence, further studies will have to decipher the respective roles of Hnf-1β in injury and regeneration.

Verdeguer *et al.* showed that ischemic AKI in mice with conditional renal-specific invalidation of *Hnf1b* in adulthood induces a severe tubular disorder characterized by the lost of planar cell polarity and abnormal epithelial cell proliferation with subsequent cystic dilatation [31]. Hnf-1β may drive the tubular regeneration by controlling the network of epithelial genes that have to be early re-expressed after mitosis. This finding was identified *in vitro* with conditions mimicking physiological process of cell renewal. The consequences of (ischemic) AKI on *Hnf1b* expression and role were not reported in this article. In a rat model of cisplatin- or gentamicin-induced renal failure, Xu et al. showed that *Hnf1b* expression is reduced three days after nephrotoxicants injection [32]. However, the characteristics of this model (i.e. induction of complete proximal tubule dysfunction leading to a renal Fanconi syndrome phenotype) precluded any analogy concerning the role of the Hnf-1β transcription factor during renal repair following ischemic insult.

In our study, we identified a more complex role of *Hnf1b* after ischemic AKI. First, during the early steps of epithelial repair, its expression is significantly decreased which is consistent with the concomitant dramatic up-regulation of *Socs3*, a gene negatively regulated by Hnf-1β [16]. *Socs3* acts as an inhibitor of signaling pathways downstream of the interleukine-6-receptor and the EGF/HGF receptors, two pathways actively involved in renal repair [10], [33], [34], [35]. Thus, one of the roles of Hnf-1β during renal epithelial repair could be to control a number of cytokines or growth factor signaling pathways by allowing the transient up-regulation of *Socs3*. Second, the subsequent transient over-expression of *Hnf1b* was accompanied by the progressive normalization of the expression of its target genes *Cdh16* and *Pkhd1*. Hence, together with the report from Verdeguer *et al* [31], our findings also confirm the role of Hnf-1β in the epithelial renal repair following ischemic AKI through the tight regulation of a number of cystic disease-associated genes that control renal tubular morphogenesis.

The molecular mechanisms that underlie the kinetics of Hnf-1β expression after AKI remain unknown. The sharp decrease suggests a direct post-transcriptional effect involving an as yet unknown pathway. We hypothesized that hypoxia, inflammatory response or growth factors may trigger the expression of *Hnf1b in vivo.* Relevant to the first possibility, we assessed the expression of Hnf-1β in a proximal tubule cell line (HK-2 cells) grown in various conditions of hypoxia. *In vitro*, hypoxia was followed by an early up-regulation of Hnf-1β (one hour), while Hnf-1β down-regulation was observed only if hypoxia was maintained for 48 to 72 hours. This effect of hypoxia was reversible as Hnf-1β expression returned to a basal value after 24 hours of normoxia. Interestingly, we demonstrated that Hnf-1β is controlled at both mRNA and protein level by hypoxia. The timing of Hnf-1β up-regulation was similar to that of Hif-1α over-expression. Hif-1α is a hypoxia-inducible transcription factor dramatically up-regulated during cell hypoxia (as soon as 0.5 to 1 hour after the start of hypoxia). Under normoxic condition, hydroxylation of Hif-1α by specific prolylhydroxylase 1–3 (PHD) leads to its degradation through the proteasomal pathway. Hypoxia inhibits Hif-1α specific PHD and abolishes the degradation of Hif-1α which is thus translocated to the nucleus where it acts as a transcription factor. During hypoxia, Hif-1α directly regulates the expression of specific target genes, like the vascular endothelial growth factor (VEGF) [36], and induces a dedifferentiation of epithelial cells with loss of epithelial markers like E-cadherin [37]. Herein, we showed that up-regulation of Hnf-1β is a very early event during hypoxia but is not dependent on Hif-1α expression. Further studies will have to better delineate the molecular pathways that regulate *Hnf1b* expression during epithelial hypoxia.

Interestingly, our *in vivo* study showed that epithelial repair is accompanied by modulation of *Hnf1b* expression potentially allowing specific transcriptional changes like transient *Socs3* over-expression. However, contrasting with *in vitro* data, first Hnf-1β change observed after ischemic insult was a dramatic fall despite the concomitant low intra-renal oxygen partial pressure observed in this model, as demonstrated by pimonidazole staining [38]. These conflicting results suggest that the expression of *Hnf1b* during epithelial regeneration is dependent of various pathways and that hypoxia is not the main regulator of *Hnf1b* in this context. Moreover, we showed that lack of Hnf-1β expression during hypoxia *in vitro* is associated with partial loss of epithelial phenotype of HK-2 cells and a trend in better survival to hypoxic stress (Figure 7). Hence, we concluded that, *in vivo*, alternative pathways favor transient Hnf-1β down-regulation in order to allow surviving epithelial tubular cells to enter in an epithelial-mesenchyme-epithelial cycle, the later being required for proper epithelial reparation [3].

Altogether, these results suggest that (1) hypoxia may have short and long term effect on *Hnf1b* expression, (2) molecular mechanisms underlying *Hnf1b* changes observed during epithelial renal repair are more complex than a sole response to hypoxia/normoxia condition and (3) *Hnf1b* may drive an adaptative response to epithelial injury by regulating growth factors/cytokine signaling (*Socs3* expression) and epithelial differentiation (*Cdh16*, *Pkhd1, Cdh1* and *Zo-1* expression). Whether these findings may be extended to other cause of acute kidney injury (*i.e.* septic or toxic insult) and to other ischemic epithelial insults (*i.e.* liver or gut injury) needs to be addressed.

## Materials and Methods

### Hemorrhagic Shock Protocol

Validation of this model of ischemic AKI was recently reported [38]. Animal experimentations were performed according to national and institutional animal care and ethical guidelines, and were approved by local board (Comités régionaux d'éthique en matière d'expérimentation animale, INSERM, Toulouse, France). Briefly, a 2 hours-hypotension (35 mmHg mean arterial blood pressure) induced by temporary blood removal was applied to C57/Bl6 female mice (Harlan France, Gannat, France), further called shocked mice. Anesthesia was based on ketamine and xylazine (250 mg/kg and 10 mg/kg, respectively). Mice were intubated and left jugular vein and femoral artery were catheterized. Mechanical ventilation through intratracheal canula was realized with a specific ventilator Minivent 845^©^ (Hugo Sachs Electronik, Germany). At the end of that period, shed blood and lactated Ringer’s solution (twice the shed blood volume) were infused in order to provide adequate fluid resuscitation. Sham-operated mice (further called sham mice) underwent the same anesthetic and surgical procedures, but neither hemorrhage nor fluid resuscitation were performed. Shocked (n = 5–6) and sham mice (n = 4) were sacrificed at different times: 3 hours, 10 hours, 24 hours, 2 days, 6 days and 21 days after the beginning of the blood removal.

### Renal Function and Histological Analyses

Glomerular filtration rate was assessed at day 2, day 6 and day 21 by measuring the inuline clearance. Kidney samples, preserved in 10% buffered formalin, were dehydrated and embedded in paraffin. Four-micrometer sections were stained with periodic acid-Schiff. Histological changes consistent with acute tubular injury (cell necrosis, loss of brush border, cast formation and tubule dilatation) were recorded.

### Molecular Analyses

The left kidney of mice was excised, washed with 1X PBS and subsequently used for molecular studies.

Total RNA was collected with the help of the RNeasy^©^ Mini Kit (Qiagen, Courtaboeuf, France) and 1 µg of total RNA was engaged in a reaction of RT-PCR (Superscript II Rnase H Reverse Transcriptase^©^ (Invitrogen, Villebon sur Yvette, France)). cDNA were subsequently quantified using the LightCycler Mix (Roche, Meylan, France) using the following conditions : 95°C for 5 min followed by 40 cycles (95°C for 15 secondes, 60°C for 10 secondes and 72°C for 15 secondes) and denaturation. Quantitative PCR was carried out on a LightCycler^©^ 480 sequence detection system. The primers were designed using Primer3 and GeneQant softwares. The efficiency of each set of primers was assessed by dilution curves and only set with efficiency higher than 95% were used. Ct differences between the reference (*Gapdh* and *18S*) and target genes were calculated for each sample. The formula used to quantify the relative changes in target over references mRNAs between the two groups is derived from the 2^−ΔΔCt^ formula as recommended. Primers used for quantitative PCR in mouse tissue were the following : *Cdh16* (D) CCAGCCTGGAGACACATACA (R) GGATCACAACAGTGGCAGAA; *Pcna* (D) AAGTGGAGAGCTTGGCAATG (R) CAGTGGAGTGGCTTTTGTGA; *Socs3* (D) GAGATTTCGCTTCGGGACTA (R) AACTTGCTGTGGGTGACCAT; *Lrp2* (D) TGGGTGTGTGACCAGGATAA (R) ACACACTGACCATTGGAGCA; *Hnf1a* (D) GAACCTCAGCCCAGAGGAG (R) GATGTTGTGCTGCTGCAAGT; *Hnf1b* (D) GCGGTGACTCAGCTACAGAA (R) CACCATTGCAGATGGGAAC; *Pkhd1* (D) AAGTCAAGGGCCATCACATC (R) ATGTTTCTGGTCAACAGCCC; *Cubln* (D) CCCTTTGGACCGTTCTGTGGCA (R) ACGGTTGCATACTCCGCATGGA.

For protein extraction, kidney samples were homogenized in lysis buffer (10 mM Tris pH 7.5, 150 mM NaCl, 1 mM EDTA, 1 mM EGTA and 0.1% SDS) with a protease inhibitor cocktail (Complete Mini kit (Roche, Meylan, France)) and separated on nitrocellulose membranes. Membranes were blocked with TBS-0.1% Tween +5% BSA at room temperature for 2 hours and probed with the monoclonal antibodies anti-Hnf-1β (sc-22840X, SantaCruz Biotechnology, Santa Cruz CA, USA; 1∶1000) overnight at 4°C. The membranes were then incubated for 1 hour with the appropriate secondary antibody (1∶7500) conjugated with peroxidase (ECL™ anti-rabbit, GE Healthcare-little Chalfont-GB). Detection was performed with SuperSignal@ West Pico Chemiluminescent Substrate (ThermoScientific, Rockford, USA) and analyzed by the photon detector Gene Gnome@ (Syngene Bio Imaging, Cambridge, UK). Membranes were reprobed with polyclonal goat anti-B-actine (1∶10 000). Protein bands were quantified by densitometry using ImageJ public open source software and results are expressed as Hnf-1β/Beta-actin ratio.

### Cell Culture

The human proximal tubule epithelial cell-line HK-2 was purchased from ATCC° (Molsheim, France) and were grown in DMEM/F12+ Glutamax° (1/1) medium supplemented with 50 U/mL Penicillin, 50 mg/mL Streptomycin, 400 µg/mL of hydrocortisone, 10 µg/mL of EGF, 1.4 µg/mL of triiodothyroxine, 5 mg/mL of insulin and 10% fetal calf serum. The cells were grown in 6-wells plates. All experiments were conducted with confluent cultures. For hypoxic culture, cells were placed in a hypoxic (1% O2, 5% CO2, 37°C) incubator (Sanyo 02/C02 incubator, Ontario) for 0, ½, 1, 2, 24, 48 or 72 h. Control cells were incubated for equivalent periods under normoxic conditions (21% O2, 5% CO2, 37°C). Last, normoxic up-regulation of Hif-1α was obtained with the use of specific prolylhydroxylase inhibitor (Calbiochem-Merck°, Fontenay-sous-bois, France) (Figure 6) or Dimethyloxallyl Glycine (DMOG, Cayman Chemical company, Ann Arbor, Michigan, USA) (Supplementary Material).

### RNA Interference

100 pmol of *HNF1B*-specific Ambion° siRNA (siRNA IS s13871, Life Technologies SAS, Saint Aubin, France) or a negative control siRNA (*Silencer*° Select negative control n°1, Life Technologies SAS, Saint Aubin, France) were transfected into HK-2 cells with DharmaFECT Duo transfection reagent (ThermoScientific, Courtaboeuf, France). After 48 hours of transfection, cells were placed into 1%-hypoxic or normoxic incubator for another 24 hours. Total RNA was collected, reverse transcripted and quantified (see among).

Primers used for quantitative PCR in human cells were the following : *HNF1B* (D) CAACCAGACTCACAGCCTGA (R) GCCATGGTGACTGATTGTTG; *CDH16 (KSP-Cadherin)* (D) TTCCCATGCCTACCTCACCTTG (R) TTGCAGCGACACACGATCACTC; *GAPDH* (D) AATCCCATCACCATCTTCCAG (R) AAATGAGCCCCAGCCTTC; *ZO-1* (D) AAGAGCACAGCAATGGAGG (R) ATTGACGTTTCCCCACTCTG; *CDH1* (*E-Cadherin*) (D) CCCAATACATCTCCCTTCACAG (R) CCACCTCTAAGGCCATCTTTG; *VIMENTIN* (D) CCAGAGGGAGTGAATCCAGA (R) AGATGGCCCTTGACATTGAG.

### Flow Cytometry Analysis

LIVE/DEAD® Fixable Dead Cell Stain Kit (Invitrogen Corporation, Carlsbad, CA USA) were used to evaluate HK-2 cell viability by flow cytometry. After treatment (siRNA transfection and/or hypoxia), cells were incubated with LIVE/DEAD® reagent for 20 min. Then, cells were analyzed by BD Fortessa flow cytometry (Becton Dickinson, San Jose, CA), as recommended by the manufacturer.

### Statistical Analysis

Quantitative parameters are reported as median and limits, and qualitative parameters as numbers and percentage. Continuous variables were compared using one-way ANOVA test followed by unpaired t-test or Bonferroni correction, as appropriated. A *P*-value <0.05 was considered as statistically significant. Analyses were performed using GraphPad Prism 4 (Graphpad software Inc, San Diego, USA).

## Supporting Information

Figure S1 **Western-blots showing kinetic of HNF-1β, HIF-1α and β-actin during hypoxia in epithelial HK-2 cells (0 to 72 hours of hypoxia).**
(TIF)

Figure S2 **Wester-Blots showing expression of HIF-1α and HNF-1β in HK-2 cells grown with a DMOG HIF-1α specific prolyl-hydroxylases inhibitor.**
(TIF)
