# Supplementary figures and images for: Hnf-1β Transcription Factor Is an Early Hif-1α-Independent Marker of Epithelial Hypoxia and Controls Renal Repair
Source: PLoS One. 2013 May 21;8(5):e63585. doi: 10.1371/journal.pone.0063585 (PMC3660442; doi:10.1371/journal.pone.0063585)

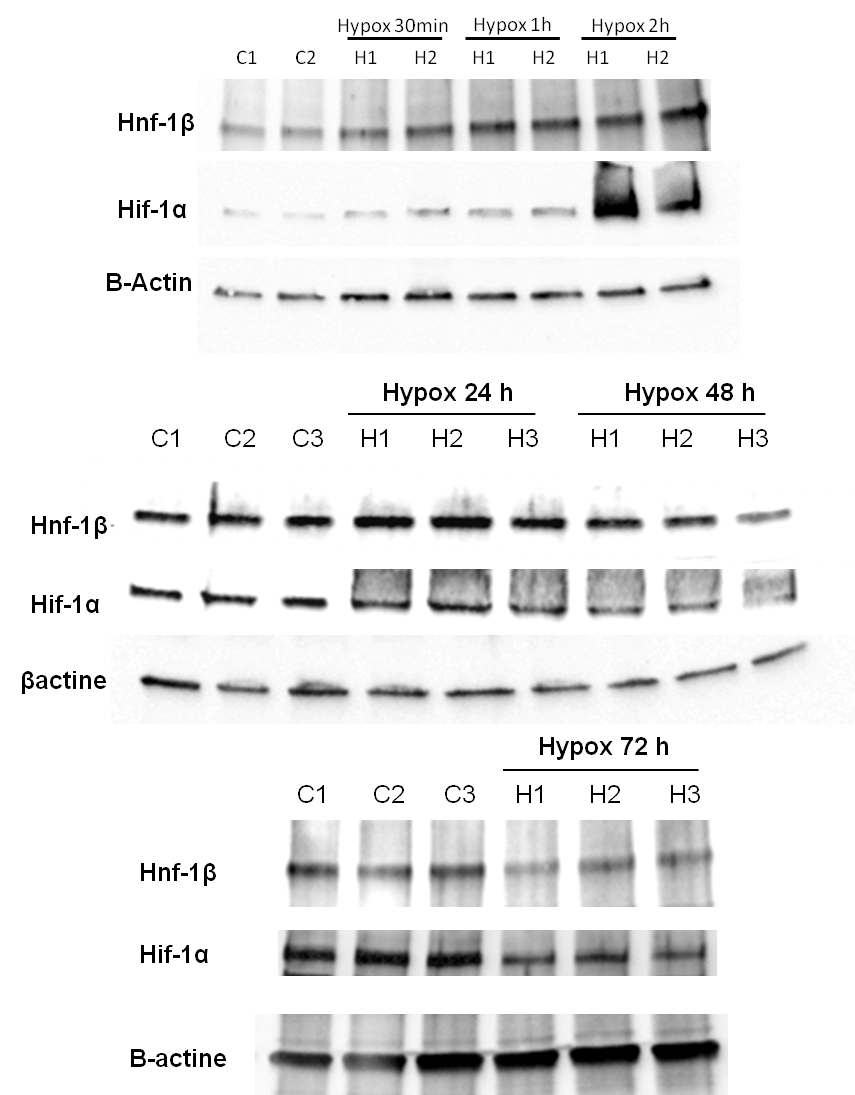

Supplement: Figure S1 — Western-blots showing kinetic of HNF-1β, HIF-1α and β-actin during hypoxia in epithelial HK-2 cells (0 to 72 hours of hypoxia). (TIF) [file pone.0063585.s001.tif]
